# In Vivo Evaluation of Injected and Bioprinted Hyaluronic Acid‐Based Bioink in Corneal Stromal Pocket

**DOI:** 10.1002/mabi.202500555

**Published:** 2026-01-18

**Authors:** Abhinav Reddy Kethiri, Paula Puistola, Maija Huuskonen, Suvi Huhtanen, Karoliina Hopia, Susanna Miettinen, Anni Mörö, Heli Skottman

**Affiliations:** ^1^ Eye Regeneration Group Faculty of Medicine and Health Technology Tampere University Tampere Finland; ^2^ Tays Eye Centre Tampere University Hospital Tampere Finland; ^3^ Adult Stem Cell Group Faculty of Medicine and Health Technology Tampere University Tampere Finland; ^4^ Tays Research Services Wellbeing Services County of Pirkanmaa Tampere University Hospital Tampere Finland

**Keywords:** corneal keratocytes, corneal stromal pocket, human adipose‐derived stem cells, hyaluronic acid bioink, multi‐material 3D bioprinting

## Abstract

The corneal stroma contains specialized stromal keratocytes (CSKs) that preserve corneal transparency and homogeneity. Stromal scarring and opacities lead to vision loss in millions globally. While corneal transplantation remains the gold standard, it is constrained by donor shortages. Cell‐based therapies using primary stromal cells show promise but still depend on donor tissue. Human adipose tissue‐derived stem cells (hASCs) offer an abundant alternative, capable of differentiating into CSKs. A three‐dimensional (3D) tissue matrix is essential for mimicking native tissue and supporting stromal regeneration. Hyaluronic acid (HA)‐based matrices emerge as promising stromal substitutes. In this study, we aim to investigate the biocompatibility of HA‐based bioink, both as injectable formulations and bioprinted constructs containing hASC‐CSKs. In vitro, bioprinted HA‐based constructs containing hASC‐CSKs exhibit high cell viability, an organized structure, and maintained transparency. In vivo, the bioink integrates progressively into the corneal stroma, considerably reducing stromal thickness within two weeks. It supports the hASC‐CSK phenotype post‐transplantation, as indicated by lumican expression. Although inflammatory responses are observed, the bioink shields transplanted cells from immune rejection, promoting graft survival and integration. These findings demonstrate that HA‐based bioink serves as a biocompatible scaffold for cell delivery, supporting stromal regeneration and highlighting its potential for future corneal therapies.

## Graphical abstract

Hyaluronic acid‐based bioinks support the viability of cells and keratocyte‐like characteristics. They integrate with host cornea stroma and adapt to irregular pockets through stable in situ gelation. The bioinks remain optically transparent, promote tissue remodeling, and reduce haze and inflammation. These findings demonstrate potential for corneal repair while protecting donor cells from early host responses.

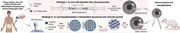

**Abbreviations**
# (numerical): animal number
3D: three‐dimensional
AS‐OCT: anterior segment—optical coherence tomography
BPC: bioprinted constructs with cells
COL1: collagen type‐1
CSK: corneal stromal keratocytes
D (numerical): day number
DMEM/F‐12: Dulbecco's Modified Eagle Medium/Ham's F12 mixture
ECM: extra cellular matrix
FGF: fibroblast growth factor
HA: hyaluronic acid
hASC: human adipose tissue‐derived stem cells
IB: injectable bioink
IBC: injectable bioink with cells
IF: immunofluorescence
KDM: Keratocyte differentiation medium
Ku80: human nuclear marker
LUM: Lumican
OCT: optimal cutting temperature compound
P: pocket control
P/S: penicillin/streptomycin
RNU: Rowett Nude rats

## 1 Introduction

Corneal opacity resulting from infections or trauma is a major cause of visual impairment affecting about 5.5 million people and ranks among the top five causes of blindness globally. While corneal transplantation remains the most effective treatment, it is affected by the shortage of donor corneas [1, 2]. Cell‐based therapies utilizing primary human corneal stromal cells have been successful [3, 4] but possess risks to living donor cornea as the harvested tissue may lose functionality or may have insufficient number of required cells [5]. Alternatively, adipose tissue is an abundant and easily accessible cell source, offering high yields of human adipose tissue‐derived stem cells (hASCs) that are immunoregulative [6] and efficiently differentiate also to corneal stromal keratocytes (CSKs) which makes it an ideal source of stem cells for regenerative medicine [7, 8, 9, 10]. Recently, autologous undifferentiated hASCs have been successfully employed in the treatment of corneal scarring in humans with no adverse effects, marking a significant advancement in corneal stromal regenerative therapies [11, 12] using cell injections. However, corneal stroma is a uniquely transparent connective tissue that relies heavily on the precise organization of its extracellular matrix (ECM) and collagen fibrils to maintain structural integrity and optical clarity [13]. In native cornea, CSKs embedded between collagen fibrils regulate the synthesis and complex organization of stromal ECM. During corneal injury and subsequent repair, dynamic CSK‐ECM interactions drive stromal remodeling, highlighting the interdependent relationship between cellular activity and matrix composition [14, 15]. This has prompted significant interest in the development of biomaterial solutions and tissue‐engineered corneal constructs designed to replicate the ECM structure and function of the native stroma [16]. For instance, gelatin methacrylate and decellularized ECMs have been extensively investigated [17, 18, 19, 20]. Recent progress in the design and fabrication of advanced biomaterial solutions has been comprehensively reviewed elsewhere [21, 22].

Building upon these developments, 3D bioprinting with cells has emerged as a promising additive manufacturing technique for engineering corneal tissue substitutes [23]. This approach enables the automated recreation of native corneal architecture, facilitating the reconstruction of complex cellular arrangements and spatial organization essential for functional tissue integration. A crucial factor in corneal 3D bioprinting is the use of optically transparent bioinks, typically based on hydrogels, which provide mechanical support, biocompatibility, and a suitable environment for cell viability, proliferation, and tissue integration. Hyaluronic acid (HA) is a naturally occurring polysaccharide and a major component of the extracellular matrix, widely distributed in connective tissues including the cornea. It plays a key role in maintaining hydration and structural integrity by retaining water and forming a viscoelastic niche that supports cellular adhesion, migration, and organization, facilitating complex tissue architecture [24]. In the cornea, HA is enriched in the limbal region and underlying stroma, supporting the limbal epithelial stem cell niche [25, 26]. After injury, HA expression has been shown to increase and contribute to the provisional extracellular matrix, where it promotes corneal epithelial repair by enhancing cell migration, upregulating repair pathways, and suppressing inflammation in both in vitro and in vivo studies [27]. Beyond its biological functions, HA offers transparency, biocompatibility, and tunable chemistry, allowing precise control over mechanics and degradation for safe tissue integration. Overall, HA is particularly advantageous in providing optimal balance between biological functionality and optical performance, making it widely employed in corneal repair and regenerative scaffolds [28, 29, 30].

In this context, we have previously reported chemically modified HA‐based bioinks with hydrazone crosslinking that form without any additional crosslinking factors a mesh‐like network, enhancing cytocompatibility and supporting printability and tissue formation post‐printing [31, 32, 33]. Furthermore, we have previously encapsulated hASCs and hASC‐CSKs within these bioinks and observed high cell viability in vitro [31, 34, 35].

Given the highly organized and heterogeneous microarchitecture of the corneal stroma, engineered constructs are designed to provide both mechanical robustness and cellular support, a criterion that makes multi‐material bioprinting particularly suitable. By combining materials with distinct stiffness and cellular compatibility, this approach enables the fabrication of bioinks that maintain biocompatibility, mechanical integrity, and a supportive environment for cell viability. Multi‐material bioprinting is already widely recognized in tissue engineering for replicating layered and complex tissues such as skin and cartilage [36]. In our previous work, we demonstrated the strategy of multi‐material corneal bioprinting using alternating cell‐laden and acellular bioinks arranged in perpendicular layers. The design improved cell proliferation and mechanical stability and showed promising ex vivo integration into corneal tissue [34, 37].

For effective therapeutic application of stromal cell therapy, selecting an optimal method for cell delivery to the cornea is critical. Various strategies have been explored, including topical application, systemic administration, subconjunctival injection, and direct intrastromal delivery [38]. Among these, intrastromal injections have shown superior outcomes, with evidence of improved corneal clarity [39, 40] and successful application in clinical cases such as keratoconus [11, 41]. Additionally, the use of bioengineered scaffolds embedded with stromal cells and implanted into the corneal stroma has demonstrated promising results in supporting cell survival, integration, and maintenance of keratocyte phenotype [17, 42].

In this study, we aim to investigate the in vivo biocompatibility of HA‐based bioink, both as injectable cell carrier formulations and bioprinted constructs containing hASC‐CSKs. For transplantation experiments, corneal stromal pockets were surgically created in rat models to facilitate either intrastromal injection of the bioink with cells or implantation of the bioprinted, cell‐laden constructs. As a result, the injected bioink crosslinked efficiently under physiological conditions and retained its structural integrity in vivo. Notably, bioprinted constructs maintained good transparency, supported high cell viability, and tissue integration. These results demonstrate the therapeutic potential of HA‐based bioinks serving as a biocompatible scaffold for corneal stromal cell delivery using both cell injection and 3D bioprinting approaches.

## 2 Results

### 2.1 hASCs Differentiated to CSKs With Stromal Cell Characteristics

**FIGURE 1:**
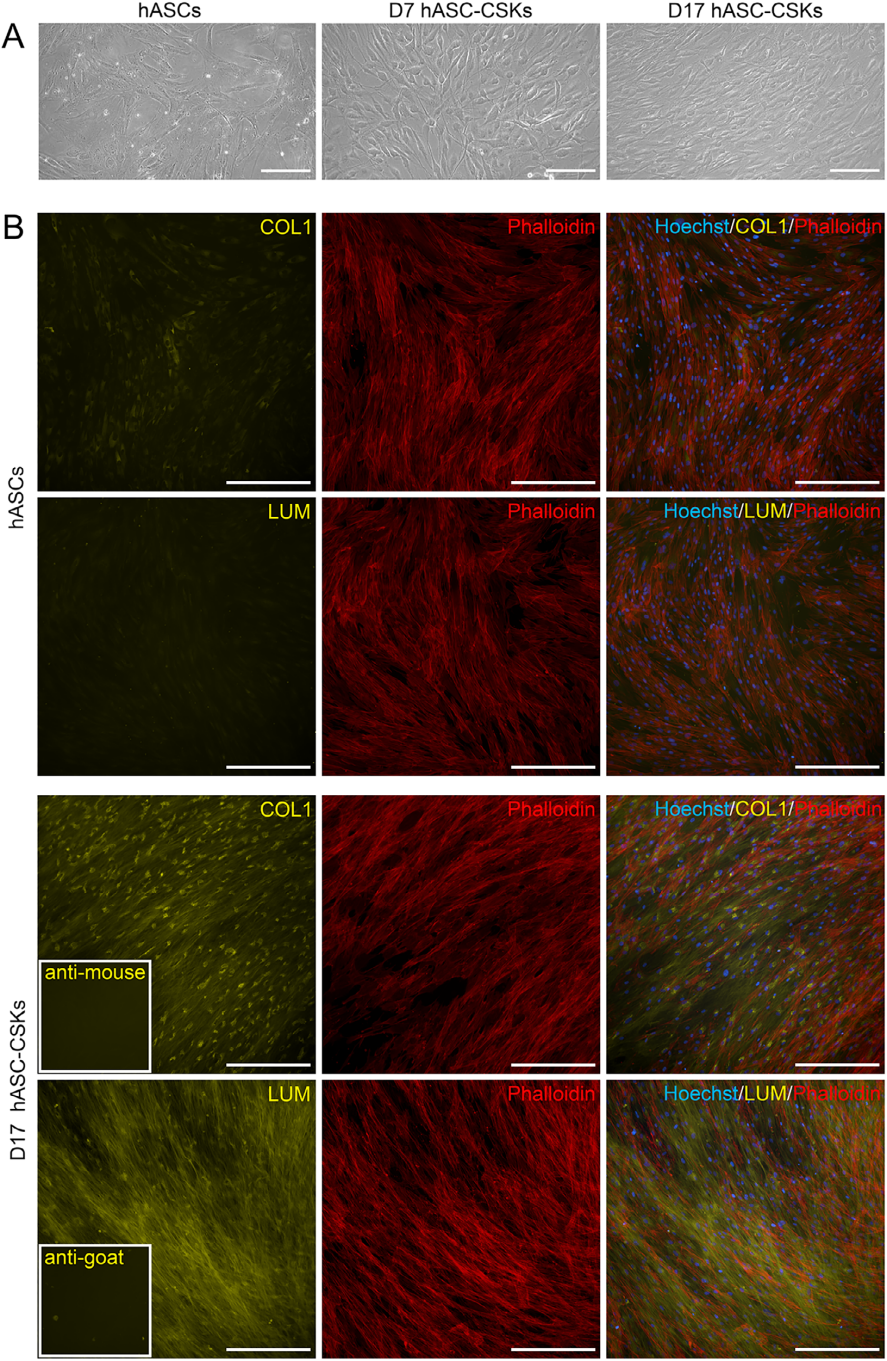
Characteristics of hASCs differentiated to CSKs. (A) Phase contrast images of hASCs, hASC‐CSKs differentiated for 7 days (D7), and hASC‐CSKs differentiated for 17 days. (B) Immunofluorescence (IF) staining of hASCs and hASC‐CSKs differentiated for 17 days (D17). The differentiation was evaluated based on the expression of COL1 (yellow) and LUM (yellow). Phalloidin (red) was used to stain actin filaments, and Hoechst (blue) to stain cell nuclei. Inset images indicate the secondary antibody controls. Scale bars: 200 µm.

First, hASCs‐CSKs were differentiated in keratocyte differentiation medium (KDM), and cells reached confluency in 6 days of culture. Phase‐contrast microscopy revealed the characteristic morphology of CSKs by day (D) 7, including rounded cell bodies and extended cytoplasmic projections, with a further increase in cell density observed by D14 (Figure 1A). After differentiation, hASC‐CSKs exhibited expression of stromal markers, including collagen type I (COL1) and lumican (LUM), with visible ECM fiber deposition. Phalloidin staining confirmed the cytoskeletal organization and cellular morphology (Figure 1B).

### 2.2 Bioprinted Structures have High Transparency and Excellent Cell Viability In Vitro

**FIGURE 2:**
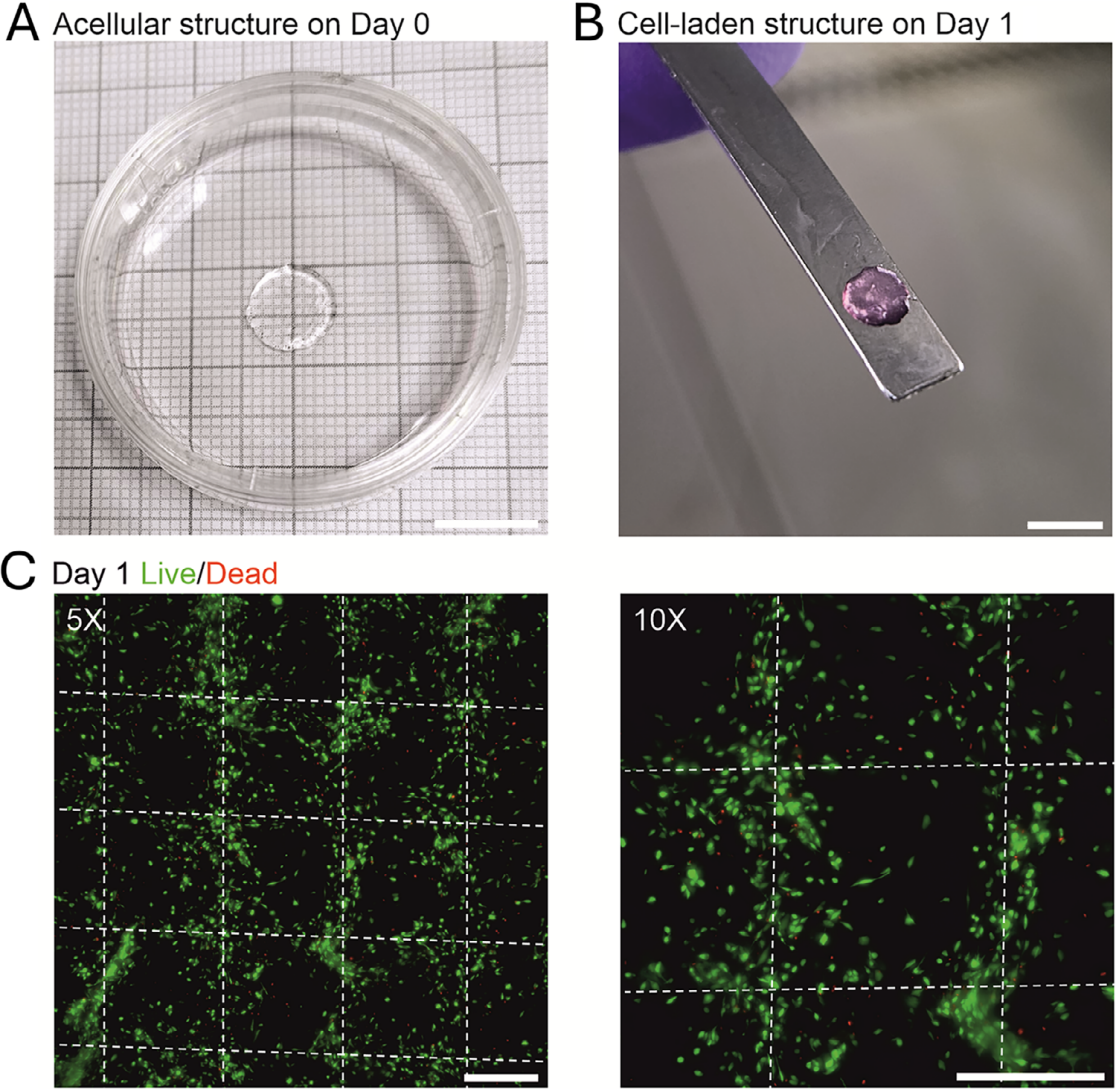
Multi‐material 3D bioprinted structures. (A) Representative acellular structure was highly transparent post‐printing. (B) A smaller cylindrical piece was punched from the bioprinted structure before transplantation. The structure had become visibly pink after incubation in cell culture medium overnight. c) The viability of d7 hASC‐CSKs (green) was good on day 1 post‐printing, and cell morphology was changing from rounded toward more elongated (*n* = 3). White dotted lines indicate hASC‐CSKs in the cell‐laden bioink showing filament‐like organization achieved by multi‐material bioprinting. Scale bars: A,B) 10 mm and C,D) 400 µm.

Given that transparency is a critical attribute in corneal bioprinting, the optical clarity of the multi‐material structures was initially demonstrated with acellular structures using acellular bioink and cell‐laden bioink (Figure 2A). However, following overnight immersion in KDM, the bioink construct exhibited a pink coloration due to the presence of phenol red in the medium (Figure 2B). Importantly, the structures were mechanically stable for handling and provided a beneficial 3D environment for the cells. Next, the viability assessment of D7 hASC‐CSKs on D1 post‐printing confirmed excellent viability as only a few small red cells were observed in the live‐dead cell assay (Figure 2C). Notably, cell morphology was already changing from rounded toward more elongated cell phenotype, indicating high cell survival from the bioprinting process and supportive 3D environment post‐printing. As multi‐material bioprinting of cell‐laden and acellular bioinks with different stiffnesses was used, the filament‐like organization of alternating bioinks was visible (Figure 2C). While the softer cell‐laden bioink protected hASC‐CSKs during bioprinting process and provided a supportive 3D environment for their growth, the stiffer acellular bioink enhanced the mechanical stability in handling and transplantations.

### 2.3 HA‐Based Bioink Promotes Early Stromal Remodeling Evidenced by Corneal Thinning In Vivo

**FIGURE 3:**
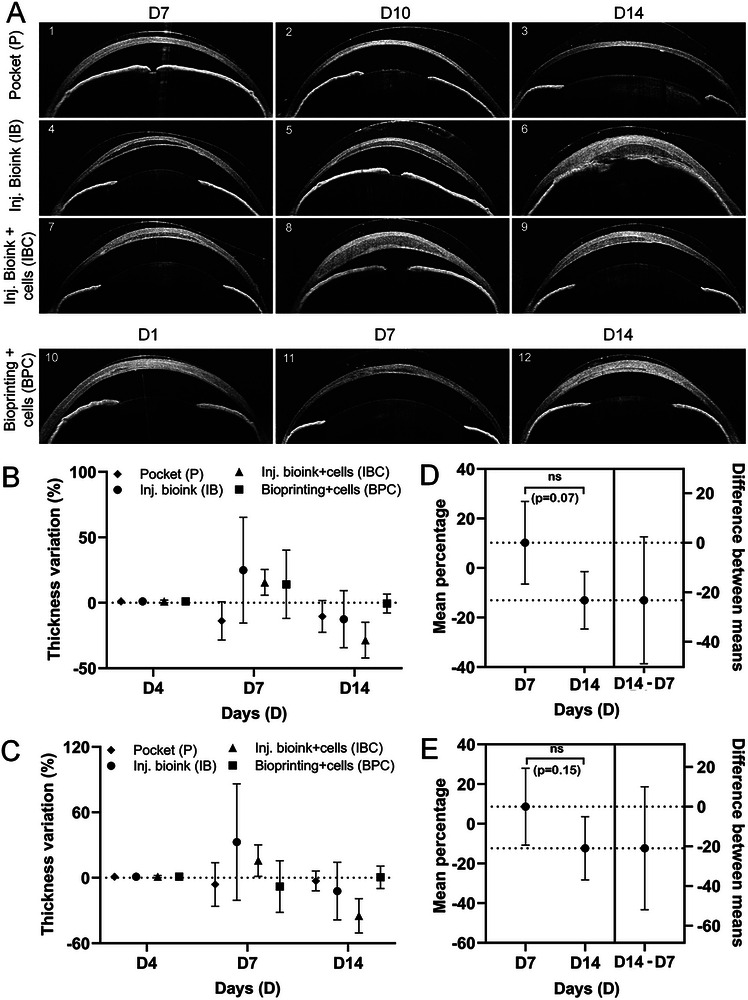
AS‐OCT and pachymetry analysis. (A) Representative AS‐OCT images showing the anterior segment of each rat eye at their respective final time points. In the control group (P; animals #1–3), gradual recovery was observed from D7 to D14. In groups with bioink injections, corneal thinning and recovery were evident both without cells (IB; animals #4,5) and with cells (IBC; animals #7–9). One exception was animal #6 (IBC), in which bioink leakage into the anterior chamber was observed. In the BPC group, recovery was visible by D1 and a thin bioink layer by D7 and D14 (animals #10–12). Longitudinal AS‐OCT imaging of the animals across all time points is provided in the supporting figure (Figure S2). (B, C) Mean percentage change in corneal (B) and stromal (C) thickness from D4 to D7 and D14, showing a reduction in all bioink‐treated groups. The control group exhibited early thinning by D7 that remained stable through D14. (D, E) Mean percentage differences in corneal (D) and stromal (E) thickness between D14 and D7 were negative across all groups, indicating further reduction in thickness by D14. However, the changes were not statistically significant. Data were obtained from *n* = 3 animals per group, with one animal analyzed at each time point. Statistical analysis was performed using unpaired t‐tests with Welch's correction, and a significance threshold was set at *p* < 0.05.

To evaluate in vivo biocompatibility, a total of 12 animals were randomly assigned to four experimental groups (*n* = 3 in each group): pocket only control (P), injectable bioink (IB), injectable bioink with cells (IBC), and bioprinted constructs with cells (BPC). Endpoint assessments were performed on D7, D10, and D14 for the P, IB, and IBC groups, and on D1, D7, and D14 for the BPC group, with one animal analyzed at each time point. Prior to transplantation, the corneas appeared normal without any abnormalities. A corneal stromal pocket was created in all animals by gently advancing a blunt spatula between the stromal layers to mechanically separate them, either for surgical control (animals #1–3) or for bioink transplantation (animals #4–12). Control animals recovered quickly with no signs of complications and appeared normal by D7 as shown in anterior segment optical coherence tomography (AS‐OCT) imaging (Figure 3A; animal #1). A representative AS‐OCT of the untreated eye is shown in Figure S1A.

HA‐based bioinks were either injected manually with a blunt needle (IB, IBC groups) or gently placed into the corneal stromal pocket using a spatula (BPC group). AS‐OCT imaging confirmed corneal recovery in groups receiving bioink injections, both without cells (IB; animals #4,5) and with cells (IBC; animals #7–9). In one eye (animal #6) bioink leaked into the anterior chamber while injecting, causing the iris remained attached to the posterior cornea. However, the cornea appeared normal on D14 (animal #6) (Figure 3A). Similarly, in the BPC group, cell‐laden constructs were successfully transplanted and integrated into the stroma, forming thin, transparent layers. The cornea of these animals showed recovery by D1 (animal #10), with a thin bioprinted structure visible on D7 (animal #11) and D14 (animal #12) (Figure 3A). These observations were further supported by longitudinal AS‐OCT imaging of all animals (Figure S2). Additionally, the bioprinted constructs appeared hypo reflective in vitro prior to transplantation, consistent with their optical transparency (inset image; Figure S2D).

Corneal and stromal thickness changes were quantified by AS‐OCT pachymetry, with negative percentage variations indicating corneal recovery and remodeling. Between D4 and D7, all bioink‐treated groups (IB, IBC, and BPC), exhibited increased corneal thickness, likely reflecting temporary stromal swelling during the early postoperative phase (IB: 24.9% ± 40.4, IBC: 15.6% ± 9.8, BPC: 14.1% ± 26.1; Figure 3B). In contrast, the pocket control (P) group showed a mean decrease of −13.7% ± 14.6, consistent with natural stromal recovery in the absence of bioink. From D4 to D14, all groups showed a reduction in corneal thickness relative to D4 (P: −10.4% ± 12.1, IB: −12.5% ± 21.8, IBC: −28.6% ± 13.6, and BPC: −0.6% ± 7.2). Stromal thickness also declined in all groups by D14 (Figure 3C). Comparison of D14 versus D7 showed mean percentage differences of −23.2% ± 10.2 in corneal thickness and −20.9% ± 12.5 in stromal thickness, suggesting bioink thinning and ongoing tissue remodeling (Figure 3D,E). Notably, the magnitude of reduction was comparable between corneal and stromal compartments, reflecting consistent thinning across tissue layers (Figure S3). Although these changes were not statistically significant, the downward trend in thickness may indicate biologically relevant tissue responses, particularly in the context of early‐stage remodeling.

### 2.4 HA‐Based Bioink Allows In Vivo Corneal Integration With Declining Inflammation and Haze

**FIGURE 4:**
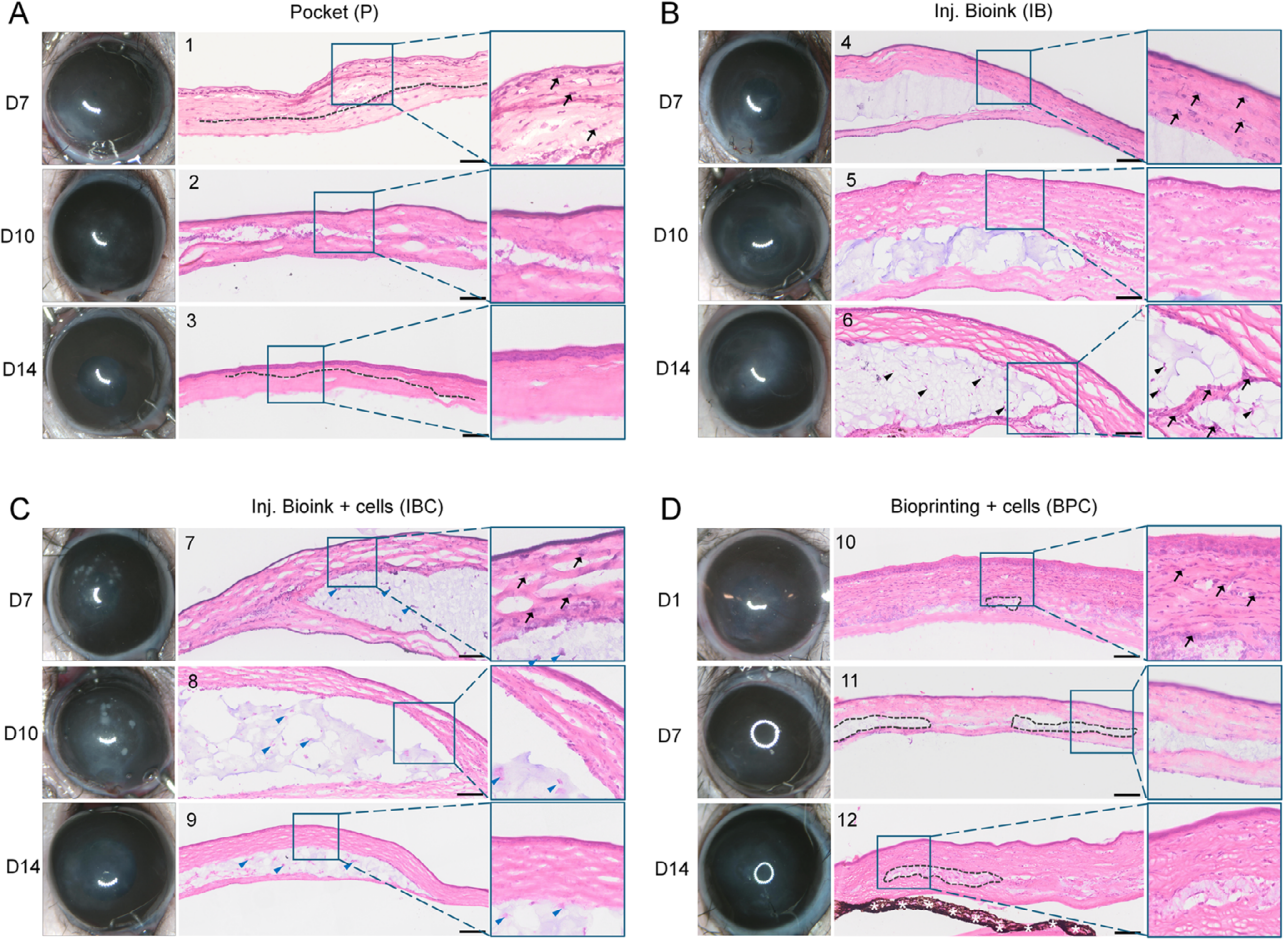
Ocular appearance and histological analysis of rat eyes. (A–D) Representative clinical images show the ocular surface of all animals at their respective end points, with varying degrees of corneal haze. D14 eyes shows improved corneal transparency in all groups, particularly when compared to their condition on D7 in Figure S4. On D7 and D10, moderate to severe corneal haze was observed in the IB (B; animals #4,5) and IBC (C; animals #7,8) groups, and on days 1 and 7 in the BPC group (D; animals #10,11), whereas the pocket‐only control group (A; animals #1,2) exhibited only mild haze. Histological analysis revealed the presence of bioink (blue/purple) sandwiched between stromal layers (pink) in all bioink groups (B, C, and D), and a distinct stromal pocket in the control group (A). Host inflammatory response was evident in early post‐transplantation stages (D7 for P, IB, and IBC; D1 for BPC), marked by increased cellular infiltration (black arrows in magnified images). However, the inflammation notably decreased on D14 in all groups (see magnified images), except for the IB group, which showed persistent inflammation in one animal (B; animal #6) due to corneal perforation during the transplantation. Grey dotted lines mark the stromal pocket or bioink region within the host tissue. Black arrowheads denote inflammatory cell infiltration, while blue arrowheads indicate hASC‐CSKs embedded in the bioink. White stars indicate iris artifacts. Data were obtained from *n* = 3 animals per group, with one animal analyzed at each time point. Scale bar: 100 µm.

Next, gross clinical examination of the operated eyes was performed to assess the progression of corneal haze over time. On D4, varying degrees of corneal haze were evident across all groups (Figure S4). By D7, the pocket control group (P) exhibited only mild haze, whereas the injection groups (IB and IBC) demonstrated moderate to high haze. In the BPC group, moderate haze was observed as early as D1, probably due to the immediate post‐implantation tissue response. However, by D14, when compared to the respective D4 images, the haze intensity visibly diminished in all the groups (Figure 4, Figure S4), indicating a trend toward corneal clarity. A notable exception was animal #6 in the IB group, which had a posterior corneal perforation during injection, resulting in persistent corneal haze through D14.

Histological analysis further revealed the presence of bioinks within the corneal stroma, identifiable as pale bluish regions distinct from the surrounding bright pink host tissue (Figure 4A–D). Additionally, inflammatory cell infiltration was evident on D7 in P, IB, and IBC groups (Figure 4A–C), as well as on D1 in the BPC group (Figure 4D), indicated by the presence of numerous nuclei within the stromal layers, an atypical feature for healthy corneal tissue (Figure S1C). By D10 and D14, inflammation visibly decreased in all groups. Quantitative assessment of nuclear density demonstrated a reduction in inflammatory cells from D10 to D14 in P and IB groups, with a statistically significant decrease in the IBC group (*p* = 0.001) (Figure S5). The BPC group displayed a slight increase in inflammatory cell density, although it was not significant. Overall, these findings indicate that the inflammatory response was primarily attributable to the surgical intervention, as also reflected in the P group. In contrast, in the eye where the bioink had leaked into the posterior chamber, persistent inflammatory cell infiltration was observed within the bioink due to posterior stromal perforation (Figure 4B; animal #6). In the IBC group, transplanted hASC‐CSKs were detectable at D7 within the compressed bioink through D14 (Figure 4C). In the BPC group, a thin bioprinted structure was observed to be integrated into the host stromal tissue (Figure 4D). The persistence of bioink in the IB, IBC, and BPC groups suggests that its degradation in rat corneas exceeds 14 days.

### 2.5 HA‐Based Bioinks Supported hASC‐CSK Characteristics In Vivo

**FIGURE 5:**
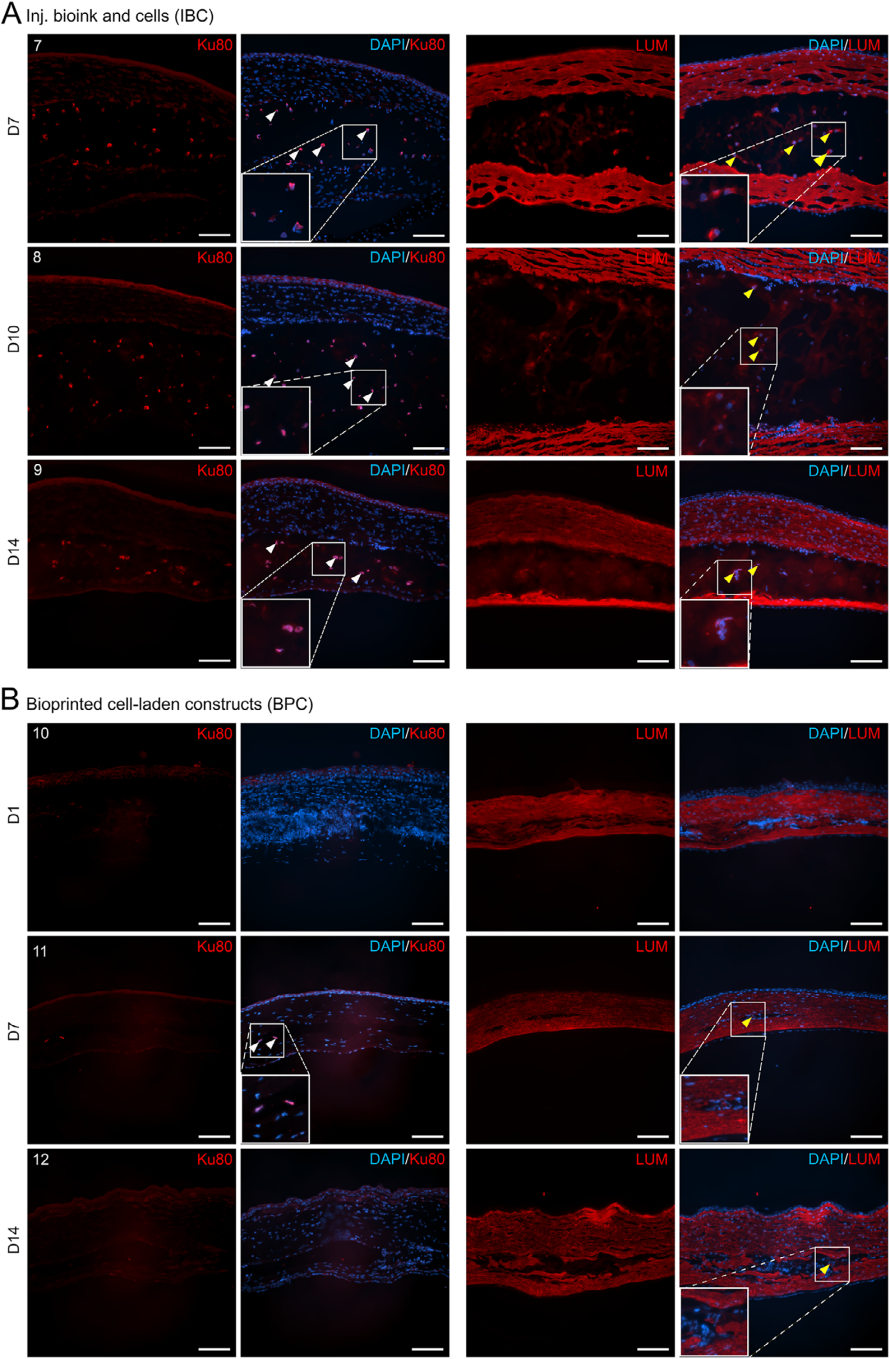
Identification and characterization of the bioink‐embedded hASC‐CSK within host corneal tissue. A‐B) Representative immunofluorescence (IF) images showing detection of transplanted hASC‐CSKs embedded within the stromal bioink. Corneal stromal marker lumican (LUM; red) stained both the host stromal tissue and transplanted hASC‐CSKs embedded in the bioink. (A) In the IBC group (animals #7–9), transplanted human cells were identified by expression of the human nuclear marker Ku80 (red) and (LUM; red). (B) In the BPC group, the smaller volume of bioprinted constructs and lower cell seeding density limited cell detectability. However, Ku80‐positive cells were detected in one animal (animal #11), and LUM expression was observed in two animals (animals #11,12). Even with limited detection, these markers indicate cell survival and maintenance of CSK phenotype in vivo. Inset images show the magnified regions. As shown in Figure S1D, normal corneal tissue does not express Ku80 but does express LUM. White arrowheads indicate Ku80‐positive cells, while yellow arrowheads denote LUM expression. Data were obtained from 2 replicates per animal (*n* = 3 animals per group), with one animal analyzed at each time point. Scale bar: 100 µm.

The ability of HA‐based bioinks to support and guide cellular behavior in vivo was evaluated in the IBC and BPC groups by tracking the transplanted hASC‐CSKs using the human‐specific nuclear marker Ku80. In the IBC group, Ku80‐positive cells were consistently detected on D7, D10, and D14, confirming the presence of the transplanted hASC‐CSKs within the bioink (Figure 4A). To further assess the maintenance of cell phenotype, lumican (LUM), a key corneal stromal marker, was analyzed. LUM staining was observed in both host stromal tissue and the hASC‐CSKs within the bioink, with transplanted cells retaining expression up to 14 days post‐transplantation and exhibiting strong expression on D7 (Figure 4A). Notably, LUM staining also revealed a morphological shift from round cell bodies on D7 to elongated cytoplasmic projections by D10 and D14, indicating active cellular remodeling and growth. Together, these findings suggest that the HA‐based injected bioink preserves cell viability, maintains hASC‐CSK phenotype, and shields transplanted cells from host inflammatory responses providing a supportive microenvironment that facilitates stromal integration (Figure 5A). In the BPC group, the smaller volume of the transplanted bioprinted cell‐laden constructs, along with a lower cell seeding density compared to the injected bioink, limited the number of detectable cells across stained sections. Nevertheless, Ku80‐positive cells were observed on D7, with some LUM‐expressing cells detectable on both D7 and D14, indicating cell survival and maintenance of CSK phenotype also in the printed structures (Figure 5B).

## 3 Discussion

Corneal stromal opacification often leads to blindness requiring transplantation, but donor shortages drive the development of transparent, biocompatible stromal substitutes for ophthalmic tissue engineering. In our previous studies, we have demonstrated the cytocompatibility and 3D printability of HA‐based bioinks [31, 32, 33, 34, 43, 44], where hydrazone crosslinking was used to avoid external crosslinkers, thereby reducing cytotoxic risks and preserving the high viability of embedded cells. Detailed synthesis methods and the physicochemical properties of these HA‐based bioinks have been described in our published work [31]. In the present study, we further evaluated the in vivo biocompatibility of the HA‐based bioink using a rat corneal stromal model. To assess the delivery efficiency and integration of cells, we employed both direct cell injection and 3D bioprinting approaches. These complementary methods allowed us to access the outcomes of conventional and biofabrication‐based delivery strategies within the same in vivo context.

Initially, in vitro analysis of the differentiated hASCs‐CSK identified LUM‐expressing cells, an important proteoglycan associated with CSKs and a typical characteristic of collagen‐1 rich ECM deposition indicating CSK characteristics [45, 46]. Given that extrusion bioprinting causes shear stress generated by the printing nozzle, which can impact cell viability [47], it was important to assess the post‐printing cell viability. The results demonstrated that differentiated hASC‐CSKs maintained high viability within the bioprinted structures, indicating minimal printing‐induced cell stress. Moreover, within one day of culture in the 3D bioprinted structures, the cells exhibited a fibroblast‐like morphology, indicating successful attachment, growth, and early signs of tissue formation. The enhanced viability and morphology of hASC‐CSKs were attributed to the use of multi‐material bioprinting, which combined mechanically stable acellular bioinks with softer, cell‐laden bioinks that provided protection against shear forces and created a supportive 3D niche for the cells [34, 37]. In vivo, the HA‐based bioinks were delivered either by injection or by manual placement of bioprinted constructs into stromal pockets surgically created using a blunt spatula to separate the corneal stromal layers. This surgical manipulation of creating the stromal pocket, particularly the use of a flattened spatula on the steep curvature of the rat cornea, likely exerted mechanical tension on the stromal lamellae, potentially inducing localized inflammation in the early postoperative period.

To monitor bioink behavior and corneal recovery, AS‐OCT was employed as the primary imaging modality throughout the follow‐up period. AS‐OCT provides high‐resolution, non‐contact cross‐sectional imaging of the anterior eye, enabling detailed assessment of corneal thickness, curvature, and reflectivity, which serve as indicators of stromal clarity, edema, or scarring. This technique allows visualization of epithelial, stromal, and endothelial layers, making it an ideal tool for detecting post‐surgical defects or assessing corneal pathologies [48]. Additionally, AS‐OCT also provides pachymetry mapping, a color‐coded topographic measurement of corneal thickness that enables detection of regional corneal irregularities [49]. In the pocket control group, animals recovered quickly post‐surgery, with AS‐OCT showing only a faint hyperreflective line at the site of the stromal dissection site by D7, and minimal corneal haze was evident in the clinical images.

Injectable HA‐based bioinks demonstrated viscosity and rapidly transitioned into stable gels within 15–20 min at room temperature, both with and without cells. While the injection volume was manually controlled, delivering precise amounts remained challenging. This limitation could potentially be overcome by optimizing gel injections with Hamilton syringes. Importantly, the injected bioink filled the irregular, non‐uniform stromal pockets created during surgery, subsequently stabilizing in place. The in situ gelation of the bioink, achieved without external crosslinking agents [33, 50, 51, 52], allows it to remain injectable and printable during application while reliably forming a stable gel upon placement. Injection of bioink, either acellular or cell‐laden, was well tolerated in all cases except one (animal #6), where posterior stromal perforation resulted in an inflammatory response and entry of the gel into the anterior chamber, as confirmed by histological analysis. In contrast, bioprinted, cell‐laden constructs formed uniform, thin, transparent layers that integrated seamlessly into the host corneal stroma, with no apparent complications. Notably, AS‐OCT imaging revealed that the bioprinted structures were hypo‐reflective both in vitro and in vivo relative to the rat cornea, indicating high optical transparency and minimal light scatter.

Pachymetry analysis further supported successful integration, showing a reduction in corneal thickness between D4 and D14 in all bioink‐treated groups. The initial increase in thickness observed up to D7 likely reflected transient postoperative stromal swelling. This was followed by a marked decrease by D14, suggesting compaction of the hydrogel and progressive remodeling of the transplanted matrix. The thinning may also be facilitated by functional endothelial activity, which could help remove excess fluid from the bioink, consistent with stromal assimilation. These findings agree with previous studies by Han et al., who demonstrated that methacrylated gelatin hydrogels were gradually assimilated by corneal stromal cells and became structurally integrated into the host tissue post‐transplantation [53]. Additionally, corneal thickness outcomes varied depending on the volume of bioink delivered to the stromal pocket, suggesting that precise volumetric control may be important for standardizing outcomes in future applications. Postoperative clinical imaging of the eye revealed varying degrees of corneal haze across the treatment groups at early time points (D7), which gradually diminished over time (D14), suggesting tissue adaptation and healing. A notable exception was one animal (#6) that experienced posterior corneal perforation, resulting in persistent haze and highlighting the impact of surgical trauma on recovery. Overall, the progressive reduction in haze supports the biocompatibility and integration of the bioink, with surgical precision being critical for optimal outcomes.

Further, the histological examination supported clinical observations. The pocket control group exhibited only a faint stromal line consistent with surgical manipulation. In the IBC group, the injected bioinks appeared stable and compacted within the stromal pocket on D14, maintaining their integrity over time. In the BPC group, the bioprinted cell‐laden construct was distinctly visible as a thin, uniform layer on D14 that was well integrated and assimilated into the host stromal tissue, indicating effective engraftment and minimal disruption to the surrounding architecture. In vivo biocompatibility and cell tracking studies identified a consistent and notable presence of Ku80‐positive cells in the IBC group. LUM‐expressing phenotype further confirmed the survival and retention of transplanted hASC‐CSKs within the stromal bioink in all eyes (animals #7–9) on D7, D10, and D14. In contrast, Ku80‐expressing cells were sparsely detected in the BPC group (animal #11), likely due to the smaller size of the transplanted bioprinted constructs and the lower cell density used during printing. The constructs included an acellular, mechanically stiffer bioink to ensure structural integrity for handling and transplantation. This design resulted in cells occupying only about half of the printed volume. Increasing the cell density within the cell‐laden bioink was not feasible with the 100 µm printing needles used, as higher densities would increase clogging risk and compromise print reliability. Maintaining cell viability under these conditions would require larger nozzle diameters, which would reduce spatial resolution. In the future, bioprinting technologies not constrained by nozzle size, such as laser‐assisted bioprinting, could enable higher cell densities without sacrificing resolution or structural fidelity. Furthermore, in the IBC group, where LUM expression was distinctively observed, a morphological transition was evident, with cells displaying a rounded morphology on D7 and elongated, dendritic‐like projections on D14, characteristic of native CSK. This morphological change is consistent with keratocyte remodeling behavior observed in physiological wound healing and aligns with earlier studies showing that encapsulated mesenchymal stem cells within permissive hydrogels (like HA and gelatin) exhibit increased cell elongation and matrix deposition over time [54]. Despite these constraints, the HA‐based bioink offers a supportive microenvironment for cytocompatibility.

Finally, this study has several limitations that should be acknowledged. The small number of animals per experimental group limits the statistical power and generalizability of the findings. This limited sample size was partly due to the technically demanding nature of manually creating stromal pockets for the rat cornea, which requires high surgical precision. Additionally, while the volume of injectable bioink (8–12 µl) was manually controlled, it was not always accurately measured, occasionally leading to excessive amounts that may have caused corneal stress. Similarly, the bioprinted constructs were small (2,3 mm in diameter) and nearly invisible to the naked eye during transplantation, even under a stereo zoom microscope, making their manual placement challenging. Overall, larger sample sizes along with large‐eyed animal models would be necessary to further strengthen the results. Inclusion of alternative biomaterial controls [55], such as fibrin glue injected into the stromal pocket, would offer a valuable comparison to better evaluate the inflammatory response and host tissue interactions relative to the HA‐based bioinks. Additionally, the relatively short follow‐up period restricts the assessment of long‐term biocompatibility, tissue remodeling, and sustained integration, warranting extended studies to fully understand the durability and safety of the bioink implants.

## 4 Conclusion

This study demonstrated that the HA‐based bioink crosslinked efficiently under physiological conditions and maintained its structural integrity in vivo. Upon injection, the bioink integrated well with the host corneal tissue, providing a supportive microenvironment for transplanted hASC‐CSKs and preserving their phenotype. This highlights its potential as a biocompatible scaffold for cell delivery. Furthermore, when applied in 3D bioprinting, the constructs exhibited good optical transparency and supported high cell viability in vitro. The bioprinted structures also integrated effectively into stromal pockets, reinforcing their suitability for corneal tissue engineering. Collectively, these findings underscore the therapeutic potential of catalyst‐free, HA‐based bioinks as versatile and biocompatible scaffolds for corneal stromal cell delivery using both cell injection and 3D bioprinting approaches.

## 5 Experimental Section

### 5.1 Ethical Statement

This study was conducted with the approval of the Regional Ethics Committee of the Expert Responsibility area of Tampere University Hospital for the extraction and use hASCs for research purposes (R15161). Written informed consent was obtained from all participants prior to sample collection. All animal experiments were conducted in accordance with the statement of the Association for Research in Vision and Ophthalmology (ARVO) for the use of animals in ophthalmic and vision research, and the methods were approved by the Project Authorization Board (ELLA) for the use of animals for scientific or educational purposes, Finland (ESAVI/45381/2022). In vivo data were reported according to ARRIVE (Animal Research: Reporting in vivo Experiments) guidelines.

### 5.2 Cell Culture and hASC‐CSK Differentiation

The isolation of hASCs from subcutaneous tissue was carried out as described previously by Lindroos et al., [56], and their surface marker profile was characterized with flow cytometry according to Kurki et al., [57]. Before differentiation, hASCs recovered from cryostock were expanded in DMEM/F‐12 (Cat# 21331020, Gibco) supplemented with 5% human serum (Serana Europe GmbH), 1% penicillin/streptomycin (P/S; Cat# 15140122, Gibco), and 1% glutamine (GlutaMAX, Cat# 35050‐038, Thermo Scientific) until desired confluency. Differentiation was initiated at passage 4 with a seeding density of 5000–7000 cells cm^−2^ and was carried out according to previously described protocols [31, 58] in KDM, consisting of Advanced DMEM (Cat# 12491015, Gibco) supplemented with 1% P/S, 1% GlutaMAX, 10 ng mL^−1^ recombinant human FGF‐basic (Cat# 100‐17A, Peprotech), 0.1 mM L‐ascorbic acid 2‐phosphate (Cat# A8960, Sigma‐Aldrich), and 1 µM retinoic acid (Cat# R2625, Sigma‐Aldrich). hASCs were differentiated for 17 days for cell injections and 7 days for 3D bioprinting, after which they were trypsinized (TrypLE Select, Cat# 12563‐029, Gibco) for bioink preparation. The earlier stage (D7) of hASC‐CSK differentiation was selected for bioprinting to maintain higher proliferative capacity and cell survival from the bioprinting process.

### 5.3 Bioink Preparation

Bioinks for cell injections and bioprinting were prepared according to the protocol for our HA‐based bioink platform [31]. The synthesis and characterization of the crosslinking components were carried out as described by Wang et al., [58]. and Koivusalo et al. [32]. For cell injections, the crosslinking components were dissolved at 18 mg mL^−1^, and cells were mixed at a density of 13–15 million mL^−1^. For bioprinting, crosslinking components were dissolved at 10 mg mL^−1^ (cell‐laden bioink) and 20 mg mL^−1^ (acellular bioink), and cells were mixed at a density of 4 million mL^−1^ to the cell‐laden bioink. The bioinks were pre‐crosslinked at room temperature before use for 10–20 min for cell injections, and for 25 min (cell‐laden bioink) or 10 min (acellular bioink) in bioprinting.

### 5.4 3D Bioprinting

Bioprinted cell‐laden structures were prepared by the multi‐material bioprinting approach described by Puistola et al. [34]. Briefly, a cylindrical 3D model (8 mm diameter, two layers of 80 µm thickness) was designed using Perfactory RP software (EnvisionTEC). The inner pattern of the model was configured in Visual Machines software (EnvisionTEC) to print alternating layers in a perpendicular orientation, with a filament spacing of 0.3 mm. Bioprinting was carried out on 35 mm Petri dishes (TC‐treated, Corning) using an extrusion‐based bioprinter 3D‐Bioplotter (EnvisionTEC). The filaments of the cell‐laden and acellular bioinks were bioprinted alternatively with 32G blunt needles (100 µm, Cellink). Printing parameters were set to 1.0–1.2 bar with a speed of 5–8 mm s^−^^1^ for cell‐laden bioink and 4.0–5.0 bar with 6–8 mm s^−^^1^ for acellular bioink. After bioprinting, the structures were stabilized for 1 h in a humid environment at 37 °C before immersing them in KDM and culturing overnight at 37 °C. Visual evaluation of the transparency post‐printing was assessed from acellular structures by imaging them against micrometer paper immediately after printing.

### 5.5 Cell Viability

Cell counter (NucleoCounter NC‐202, ChemoMetec) was used to assess the cell viability in cell injections before mixing hASC‐CSKs in the bioink. For bioprinted constructs, viability was evaluated on day 1 post‐printing (*n* = 3) using LIVE/DEAD Viability/Cytotoxicity Kit (Cat# L3224, Invitrogen) according to the manufacturer's instructions. After 30 min of incubation at 37 °C, the bioprinted constructs were imaged with a fluorescent microscope (DMi8, Leica Microsystems). The stack images were edited in Fiji‐ImageJ (ImageJ 1.52n) and presented as maximum intensity projections.

### 5.6 In Vivo Corneal Stromal Pocket Model and Evaluation

For transplantation, 6–8 weeks old, female Rowett Nude (RNU) rats (*n* = 12) were procured from Charles River Laboratories, Germany, and were maintained ad libitum. The animals were randomly assigned to four experimental groups (experimental unit; *n* = 3 per group) for transplantation: pocket only control (P), injectable bioink (IB), injectable bioink with cells (IBC), and bioprinting with cells (BPC).

Surgical procedures were performed on the left eye, while the contralateral right eye remained untreated and served as internal control. All surgical procedures were carried out by a trained corneal surgeon under general anesthesia administered via subcutaneous injection, supplemented with topical ocular anesthesia (Oxybuprocaine hydrochloride 0.4% w/v, Minims, Bausch and Lomb) in a sterile hood. Prior to surgery, corneal thickness was measured using AS‐OCT to determine the appropriate stromal depth. A peripheral incision was then made with a diamond blade micrometer knife (Micrometer AK5002, MicroSurgical Technology Inc.) set to 50–60 µm. Using a blunt corneal dissector, the stromal layers were gently separated from the incision site to create a pocket approximately 3–4 mm in diameter.

Depending on the assigned group, either the bioink (8–12 µL, with or without cells) or bioprinted cell‐laden constructs (2–3 mm diameter) were delivered into the pocket via blunt needle injection or gentle placement using a spatula, respectively. The incision site was then closed with one or two interrupted 10–0 nylon sutures, and the eyelids were left open to accommodate post‐operative ocular swelling. Postoperative care included topical application of a combined antibiotic and steroid ointment (Oftan Dexa‐Chlora, Santen) three times daily for one week. Follow‐up examinations were conducted under inhalation anesthesia using clinical imaging with a stereozoom microscope and AS‐OCT on D4, D7, D10, or D14. High‐resolution cross‐sectional scans and pachymetry maps were obtained to assess graft integration and corneal thickness. Animals with severe corneal melts were excluded. Details of follow‐up and endpoint analyses for each animal are summarized in Table 1.

**TABLE 1:** Summary of animal grouping, clinical evaluation time points, and study endpoints.

Groups	Animals	AS‐OCT/clinical image time points	End point (Histology/IF)
Pocket (P)	#1	D4 and D7	D7
	#2	D4, D7, and D10	D10
	#3	D4, D7, and D14	D14
Injection bioink (IB)	#4	D4 and D7	D7
	#5	D4, D7, and D10	D10
	#6	D4, D7, D10, and D14	D14
Injection bioink with cells (IBC)	#7	D4 and D7	D7
	#8	D4, D7, and D10	D10
	#9	D4, D7, D10, and D14	D14
Bioprinted constructs with cells (BPC)	#10	D1	D1
	#11	D4 and D7	D7
	#12	D4, D7, and D14	D14

Clinical images were used to assess the degree of corneal haze, while corneal and stromal thickness were quantitatively evaluated using pachymetry maps generated by AS‐OCT. These maps provided quantitative measurements at nine predefined locations across the corneal surface, including the central point and eight peripheral points arranged in a radial pattern. Data were blinded by randomly assigning numerical codes to each animal prior to analysis. For each animal, mean corneal and stromal thickness values were calculated on D4 and compared to values on D7 and D14 to determine percentage changes over follow‐up time. The percentage decrease in corneal thickness on D14 was also compared to D7 across all groups. Additionally, mean percentage variations in both corneal and stromal thickness were analyzed at D7 and D14.

### 5.7 Histology and Immunofluorescence

Cells were characterized by immunofluorescence (IF) for both in vitro and in vivo samples. For in vitro characterization, D17‐hASC‐CSKs were processed according to the protocol described previously by Sorkio et al. [59]. Briefly, cells were fixed in 4% paraformaldehyde (Cat# 15713‐S, 20% PFA, Electron Microscopy Sciences) for 15 min, rinsed, and blocked with 3% BSA (Cat# A7906, Sigma‐Aldrich). Samples were incubated with primary antibodies (Table S1) overnight at 4 °C along with Phalloidin‐tetramethylrhodamine B isothiocyanate (Cat# P1951, Sigma‐Aldrich, 1:800) staining to visualize actin filaments. After two PBS rinses, cells were incubated with the appropriate secondary antibody (Table S1) and Hoechst 33342 (Cat# H3570, Invitrogen, 1:3000) for 40 min, rinsed thrice with PBS, and mounted with an antifade mountant (ProLong Gold, Cat# 36930, Invitrogen).

Whole rat eyes were fixed by both perfusion and immersion in 4% PFA for 3 h, followed by three 15‐min PBS washes with gentle rocking. The tissues were dehydrated in 20% sucrose overnight at 4 °C, briefly rinsed in PBS, embedded in OCT compound, and snap‐frozen on a metal block chilled with liquid nitrogen. Cryosections (10 µm) were prepared using a cryomicrotome (CM3050 S, Leica Biosystems) and stored at −20 °C until use. For staining, tissue sections were thawed for 15 min at room temperature, rehydrated with PBS, blocked with BSA, and incubated overnight with primary antibodies at 4 °C. This was followed by incubation with secondary antibodies, PBS washes, and mounting with DAPI‐containing antifade medium (ProLong Gold, Cat# P36931, Invitrogen). Imaging was performed using a fluorescence microscope (IX51, Olympus Life Science Solutions). Tissue histology was performed under a fume hood. Cryosections were thawed and heated to 65 °C for 30 min, followed by gradual rehydration through a series of graded ethanol and distilled water washes. Slides were then immersed in Hematoxylin (Cat# 01825, Mayers HTX PLUS, HistoLab) for 1 min and rinsed under running tap water for 7 min. Subsequently, Eosin (Cat# 01650, Eosin Y 0.2%, HistoLab) staining was performed for 30 s, followed by a 2‐min rinse under running water. The slides were dehydrated through a graded ethanol series and cleared in xylene for 2 min. Finally, coverslips were mounted, and the slides were imaged using a slide scanner (SLIDEVIEW VS200 system, Olympus Scientific Solutions). Image analysis was carried out using QuPath software (v0.5.1), and nuclear density quantification [60] was carried out using Fiji‐ImageJ, as described in Experimental Method S1 and Figure S5.

### 5.8 Statistical Analysis

Statistical analysis was carried out using GraphPad Prism (v10.3.0). Normal data distribution was assessed using the Shapiro‐Wilk test. AS‐OCT pachymetry measurements were obtained from nine predefined points on each cornea, and comparative analysis was performed from the mean thickness values of all the groups (*n* = 4). Differences in corneal and stromal thickness between time points were evaluated using an unpaired *t*‐test with Welch's correction. Multiple comparisons among the groups were not feasible due to the limited sample size. Statistical significance was defined as *p* < 0.05.

## Author Contributions

Conceptualization: Abhinav Reddy Kethiri, Paula Puistola, Maija Huuskonen, and Heli Skottman. Data curation: Abhinav Reddy Kethiri and Paula Puistola. Formal analysis: Abhinav Reddy Kethiri, Paula Puistola, Maija Huuskonen, and Suvi Huhtanen. Funding acquisition: Heli Skottman, Anni Mörö, Susanna Miettinen, Abhinav Reddy Kethiri, and Paula Puistola. Investigation: Abhinav Reddy Kethiri, Paula Puistola, Maija Huuskonen, Suvi Huhtanen, and Karoliina Hopia. Methodology: Abhinav Reddy Kethiri, Paula Puistola, Maija Huuskonen, Karoliina Hopia, and Anni Mörö. Project administration: Heli Skottman. Resources: Heli Skottman, Susanna Miettinen, and Anni Mörö. Supervision: Heli Skottman. Validation: Abhinav Reddy Kethiri and Paula Puistola. Visualization: Abhinav Reddy Kethiri and Paula Puistola. Writing – original draft: Abhinav Reddy Kethiri, Paula Puistola, Maija Huuskonen, and Heli Skottman. Writing – review & editing: Abhinav Reddy Kethiri, Paula Puistola, Heli Skottman, Maija Huuskonen, Anni Mörö, Susanna Miettinen, Suvi Huhtanen, and Karoliina Hopia).

## Funding

Jane and Aatos Erkko Foundation (200063 H.S. and A.M.), Research Council of Finland (324082 A.M., 336666 S.M., 326588 S.M., 312413 S.M., 337607 S.M., 365398 H.S.), Research‐to‐Business funding by Business Finland (6763/31/2021 A.M. and H.S.), Finnish Cultural Foundation (00230595 A.R.K., 00232448 and 00242643 P.P.), and Eye and Tissue Bank Foundation (20230015 P.P.)

## Conflicts of Interest

A.M. is an inventor of a pending patent (#PCT/FI2022/050403) related to the bioink used. Based on the Act on the Right in Inventions in Finland, all authors employed by Tampere University have given all rights to the University. Tampere University has transferred these rights to LifeGlue Technologies Oy. A.M., H.S., and P.P. are co‐founders and shareholders in LifeGlue Technologies Oy. P.P. and S.H. are employees in LifeGlue Technologies Oy. The other authors declare no conflicts of interests.

## Supporting information




**Supporting File**: mabi70134‐sup‐0001‐SuppMat.pdf.

## Data Availability

The data that support the findings of this study are available from the corresponding author upon reasonable request.
