# Supplementary material for: In Vivo Evaluation of Injected and Bioprinted Hyaluronic Acid‐Based Bioink in Corneal Stromal Pocket
Source: Macromol Biosci. 2026 Jan 18;26(1):e00555. doi: 10.1002/mabi.202500555 (PMC12812298; doi:10.1002/mabi.202500555)
Supplement: Supplementary file 1 — Supporting File: mabi70134‐sup‐0001‐SuppMat.pdf. [file MABI-26-e00555-s001.docx]

Supporting Information

***In vivo* evaluation of injected and bioprinted hyaluronic acid based bioink in corneal stromal pocket**

Abhinav Reddy Kethiri, Paula Puistola, Maija Huuskonen, Suvi Huhtanen, Karoliina Hopia, Susanna Miettinen, Anni Mörö, Heli Skottman*

**Experimental method S1:**

*Quantification of total nuclear density*

Histological sections of the central and mid-peripheral cornea were acquired at 60x magnification using a high-resolution slide-scanning microscope (SLIDEVIEW™ VS200 system, Olympus Scientific Solutions). For each corneal region, three non-overlapping fields of view were captured per section to ensure representative sampling of the stromal architecture (Figure S5A). All identifiable nuclei, recognized by their characteristic purple hematoxylin staining, were manually annotated using the point-selection tool in Microsoft Paint, and the annotated images were subsequently imported into Fiji-ImageJ (ImageJ 1.52n) for quantification.

Nuclear counts for each image were quantified using ImageJ, and the total number of nuclei per section was computed (Figure S5B). To estimate inflammatory cell density, a baseline-exclusion approach applied.^[60]^ Briefly, the baseline nuclear count derived from healthy control corneas (without inflammatory infiltration) was subtracted from the total nuclear counts of experimental samples. The resulting adjusted values represented the mean inflammatory cell count for each time point. These mean values were plotted on the y-axis against the experimental endpoint day on the x-axis (Figure S5C) to assess changes in inflammatory cell infiltration.


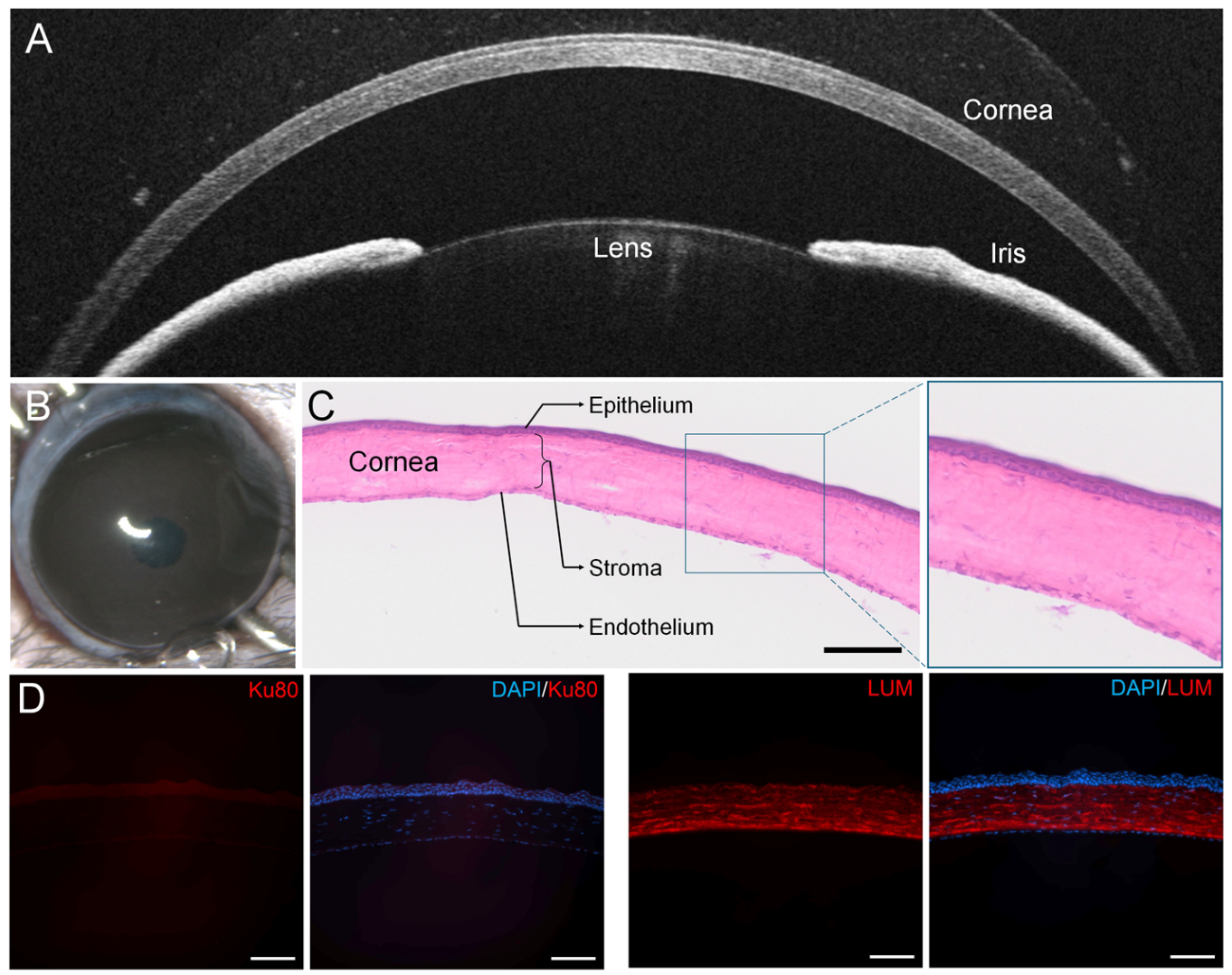


**Figure S1:** Ocular appearance, AS-OCT, and histology of the rat eye. A) Representative AS-OCT image of a normal rat eye showing a cornea at the top, with hyper-reflective flaps laterally indicating the iris, and a centrally located hypo-reflective region corresponding to the lens. B) Stereo zoom microscope image showing the gross appearance of a healthy rat eye. C) Histological section of the cornea displaying organized epithelial, stromal, and endothelial layers with magnified image. D) IF staining results indicate that human-specific Ku80 is not expressed, whereas LUM is detected in the normal cornea. Scale bar: 100 µm.


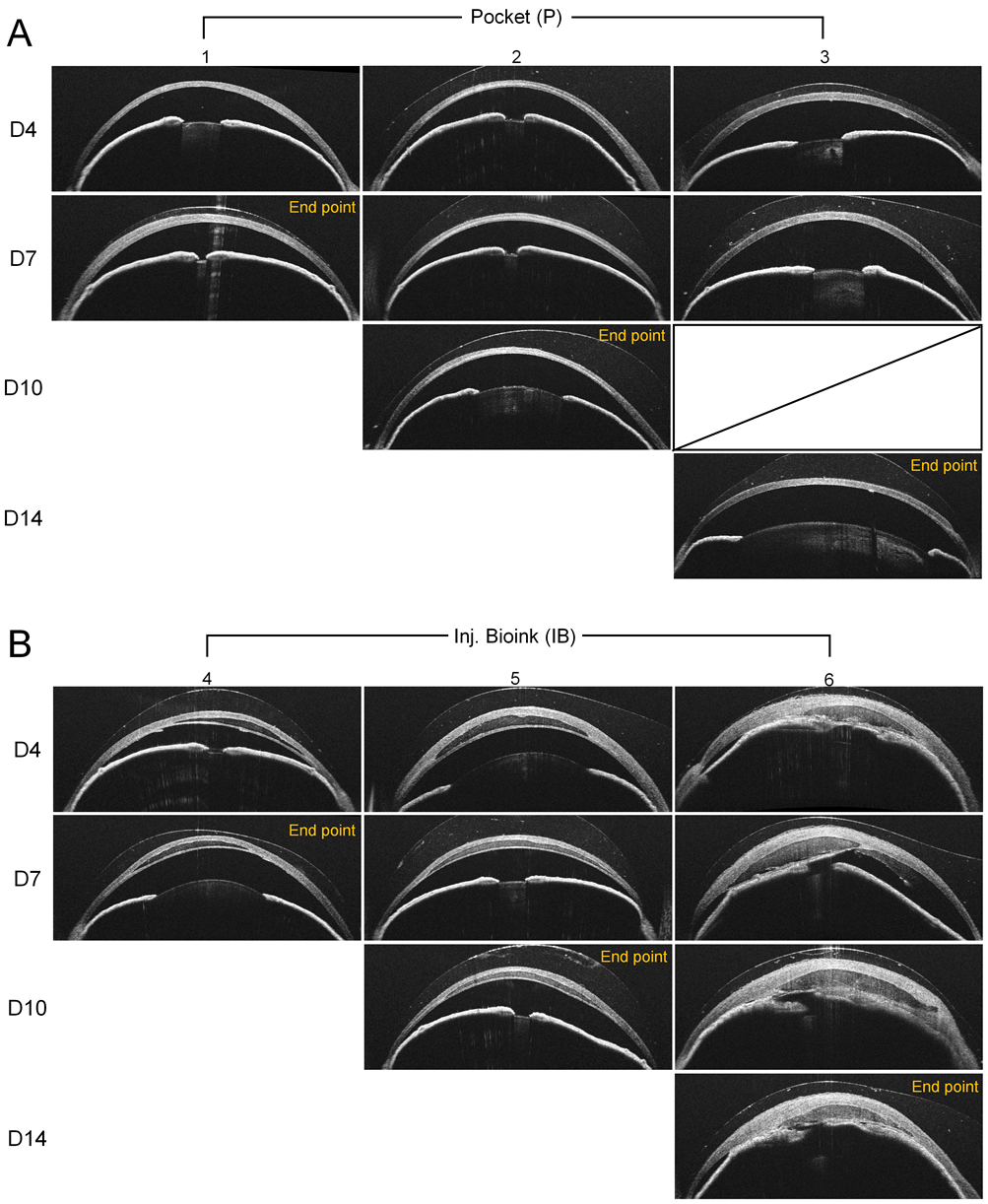


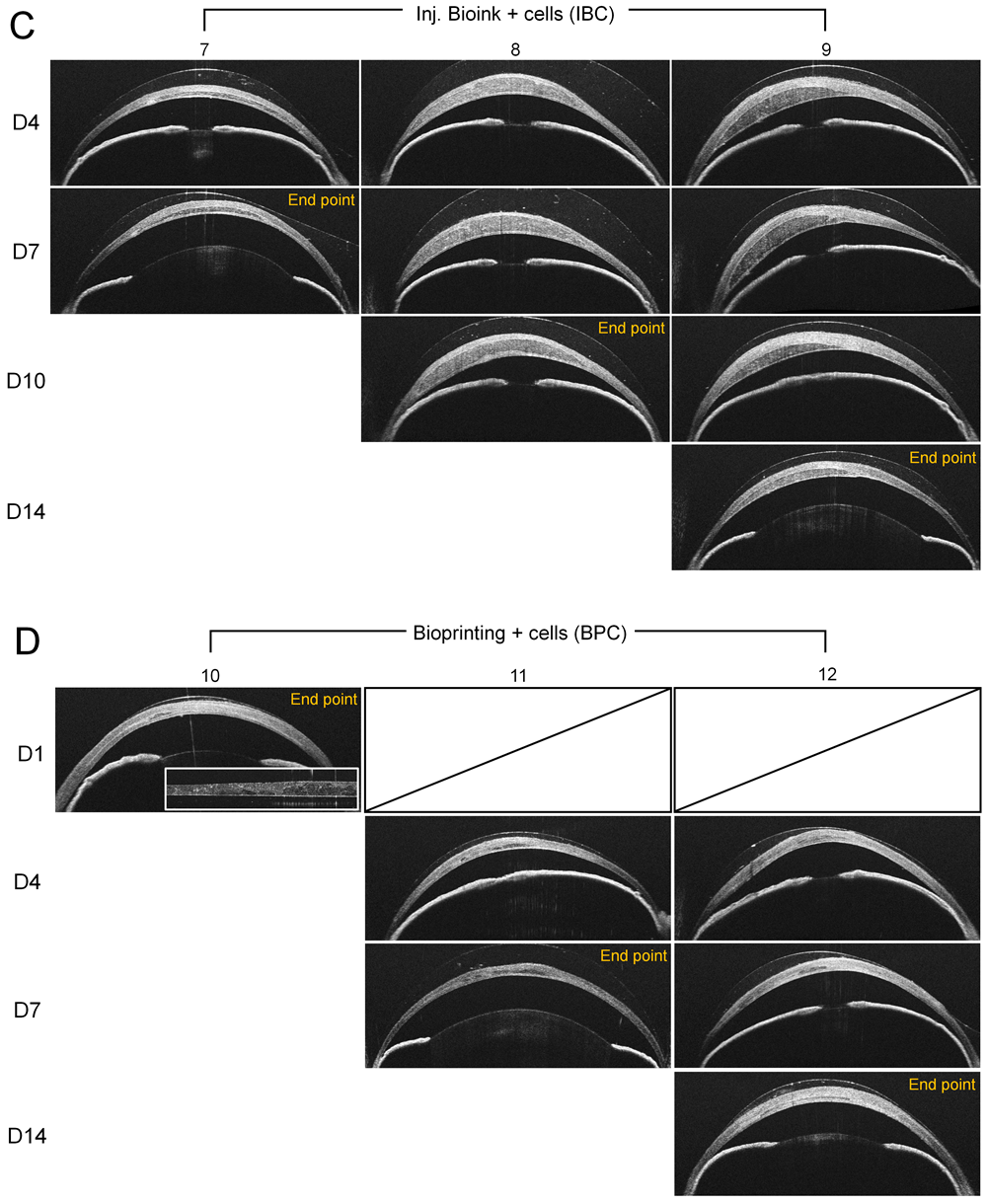


**Figure S2:** Longitudinal AS-OCT imaging. A-D) Representative AS-OCT images of the sequential follow-up of all the animals used in the study. Thin hyper reflective streaks within the stroma marked the pocket location and demonstrated progressive corneal recovery from D4 through D14 in P group (A; animals #1-3). In both the IB (B; animals #4-6) and IBC (C; animals #7-9) groups, compaction of the injected bioink was evident by D7, D10, or D14 compared with respective earlier time points. Notably, bioink leakage into the anterior chamber was observed on D7 in animal #6. In the BPC group (D; animals #10–12), thin layers of cell-laden constructs were visible but showed no marked structural changes between D4 and D14. In all groups, the bioink appeared hypo reflective relative to the host cornea. The inset shows a magnified (5x) in vitro AS-OCT image of the bioink. Box with a diagonal line indicates images were not obtained at that time point. Data was obtained from *n* = 3 animals per group, with one animal analyzed at each time point.


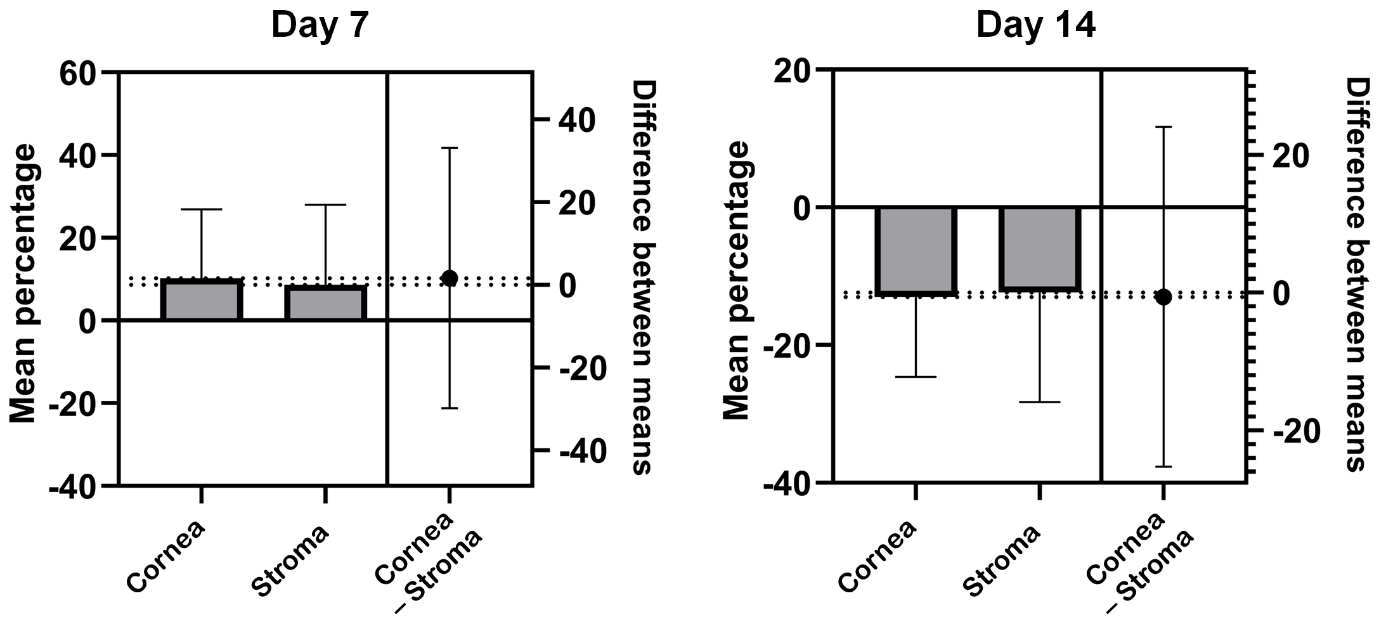


**Figure S3:** Mean differences in corneal and stromal thickness. Representative graph illustrating an increase in both corneal and stromal thickness on D7, followed by a decrease in both parameters on D14. Comparison of mean thickness changes between D7 and D14 revealed that the magnitude of change was comparable between the cornea and stroma at each time point. Data was obtained from *n* = 3 animals per group, with one animal analyzed at each time point. Statistical analysis was performed using unpaired t-tests with Welch’s correction and a significance threshold was set at p < 0.05. No statistical difference was found.


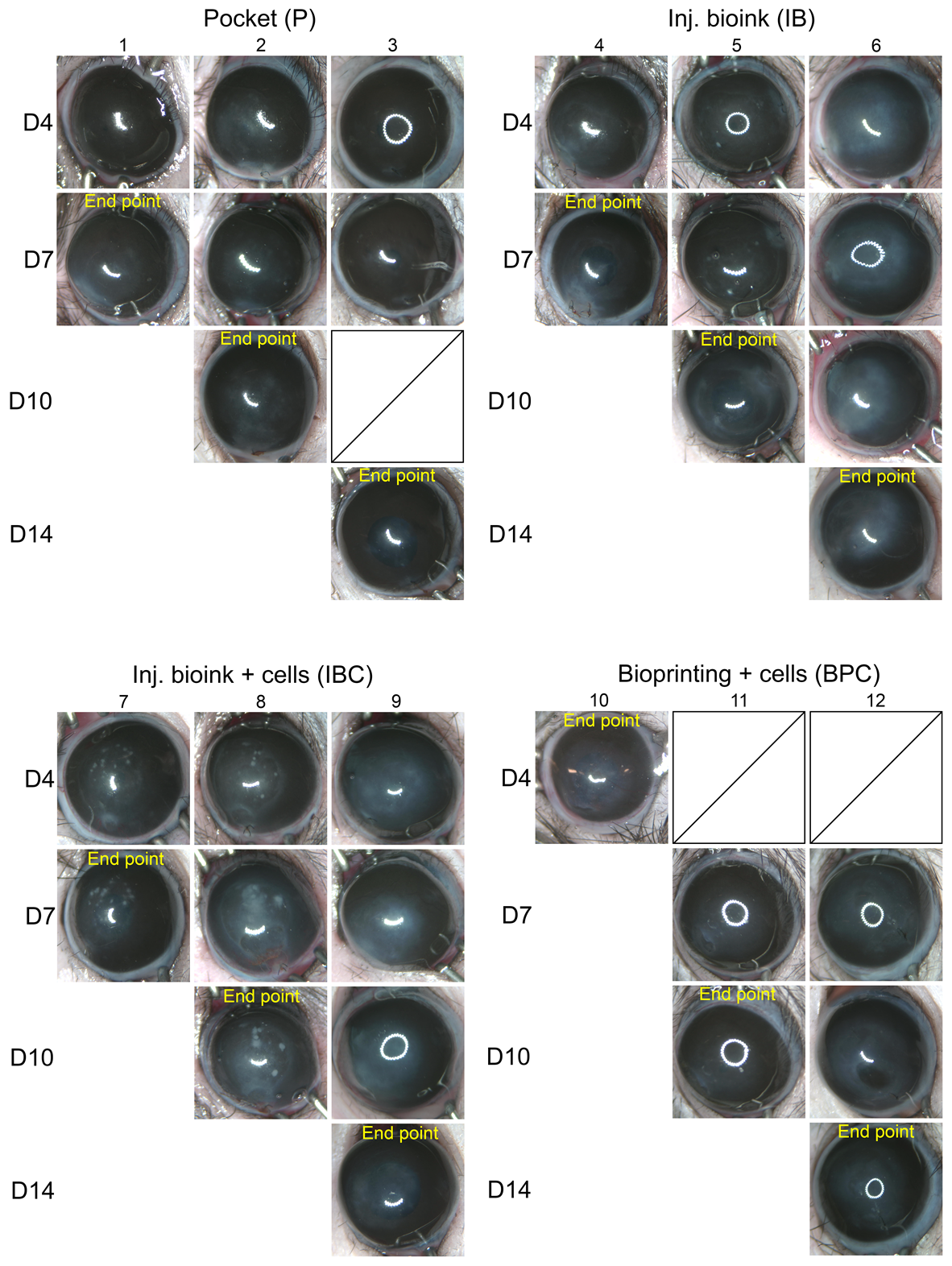


**Figure S4:** Longitudinal imaging of cornea. Representative clinical images show sequential follow-up of all animals used in the study. Varying degrees of corneal haze were observed on D4 in the P, IB, and IBC groups, and as early as D1 in the BPC group. The P group exhibited minimal corneal haze on D4, which remained stable through D14. Corneal clarity improved by D14 in both the IBC and BPC groups compared to their earlier time points. However, in the IB group, persistent corneal haze was noted in animal #6 on D14, attributed to bioink leakage into the anterior chamber. Data was obtained from *n* = 3 animals per group, with one animal analyzed at each time point.


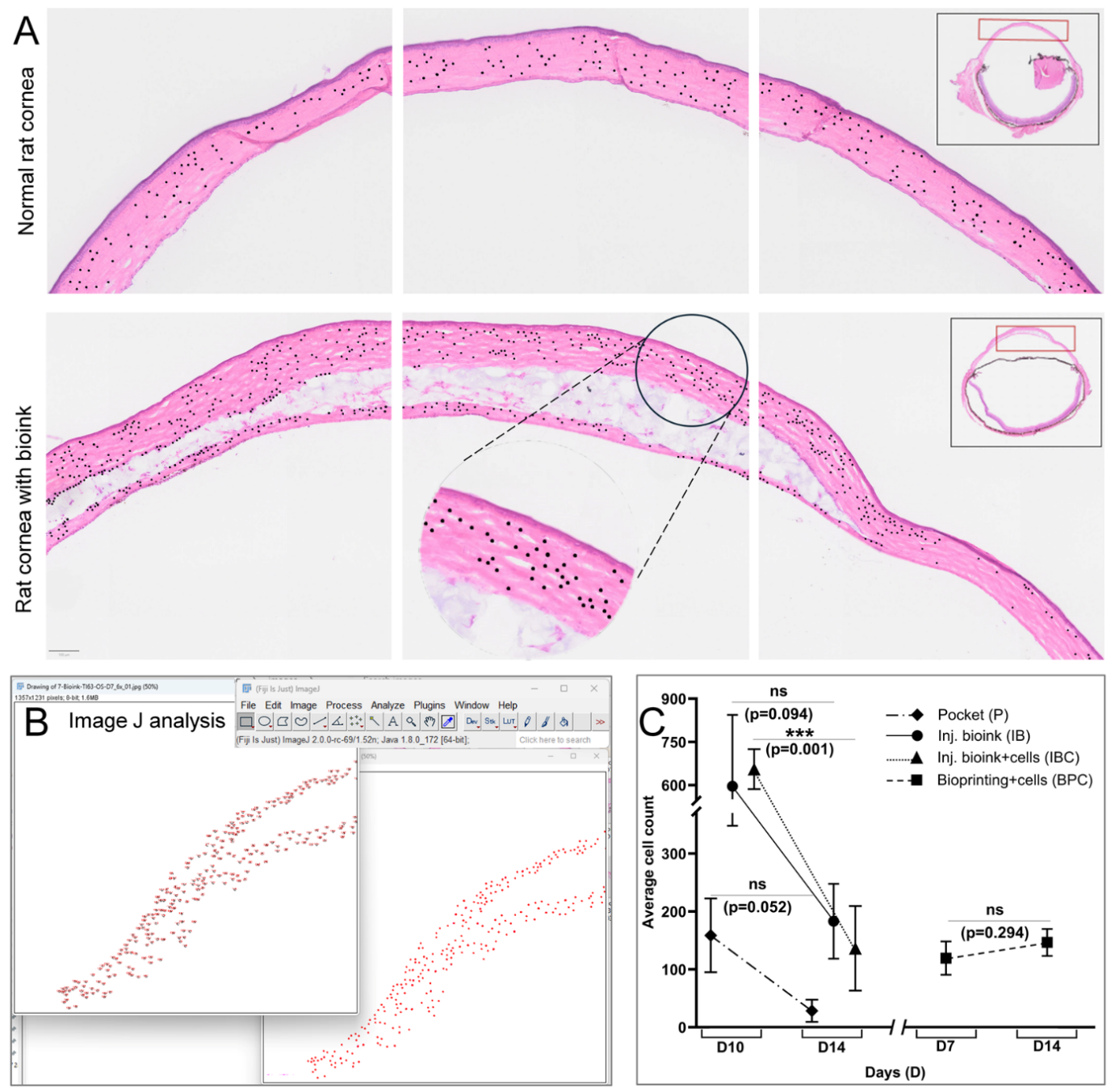


**Figure S5:** Cell density analysis. A) Histological sections of the normal rat cornea and cornea containing bioink. Three non-overlapping fields of view were captured at 60x magnification to ensure representative sampling of the stromal architecture. All nuclei were manually marked (black dots) for subsequent ImageJ quantification. B) ImageJ processing demonstrates the color-threshold adjustments to isolate and count the manually marked nuclei. C) Mean nuclear counts from histological sections demonstrated a reduction in inflammatory cell density from D10 to D14 in both the pocket (P) and injection bioink (IB) groups. In the injection bioink with cells group (IBC), a statistically significant decrease in cell counts was observed. In contrast, the bioprinting with cells group (BPC) showed a slight increase in cell density from D7 to D14, although this change was not statistically significant. Data were obtained from n = 3 animals per group, with one animal analyzed at each time point. Statistical analyses were performed using unpaired t-tests with Welch’s correction, with significance defined as p < 0.05.

**Table S1:** List of antibodies used for immunofluorescence

| **Antibody** | **Host** | **Dilution** | **Catalog/Manufacturer** |
| --- | --- | --- | --- |
| COL1 | Mouse | 1:100 | Cat# ab6308, Abcam |
| Lumican (LUM) | Goat | 1:300 | Cat# AF2846, R and D Systems |
| Ku80 | Rabbit | 1:100 | Cat# 2180, Cell Signaling Technology |
| Anti-Mouse IgG Alexa Fluor^TM^ 488 | Donkey | 1:800 | Cat# A-21202, Thermo Scientific |
| Anti-Goat IgG Alexa Fluor^TM^ 488 | Donkey | 1:800 | Cat# A11055, Thermo Scientific |
| Anti-Goat IgG Alexa Fluor^TM^ 568 | Donkey | 1:800 | Cat# A-11057, Thermo Scientific |
| Anti-Rabbit IgG Alexa Fluor^TM^ 568 | Donkey | 1:800 | Cat# A10042, Thermo Scientific |
